# Establishing a comprehensive panel of patient-derived xenograft models for high-grade endometrial carcinoma: molecular subtypes, genetic alterations, and therapeutic target profiling

**DOI:** 10.1016/j.neo.2025.101158

**Published:** 2025-04-07

**Authors:** Sho Sato, Shigehiro Yagishita, Hiroshi Yoshida, Daisuke Shintani, Aiko Ogasawara, Tadaaki Nishikawa, Masanori Yasuda, Keiji Furuuchi, Toshimitsu Uenaka, Akinobu Hamada, Kosei Hasegawa

**Affiliations:** a Department of Gynecologic Oncology, Saitama Medical University International Medical Center, Saitama, Japan; b Division of Molecular Pharmacology, National Cancer Center Research Institute, Tokyo, Japan; c Department of Pharmacology and Therapeutics, National Cancer Center Research Institute, Tokyo, Japan; d Department of Diagnostic Pathology, National Cancer Center Hospital, Tokyo, Japan; e Department of Medical Oncology, National Cancer Center Hospital, Tokyo, Japan; f Department of Pathology, Saitama Medical University International Medical Center, Saitama, Japan; g Epochal Precision Anti-Cancer Therapeutics, Eisai Inc., Exton, PA, USA

**Keywords:** Endometrial cancer, patient-derived xenograft (PDX), antibody-drug conjugate (ADC)

## Abstract

**Background:**

High-grade endometrial cancer (EC) has a poor prognosis, but molecular classification-based treatments present new therapeutic opportunities. Antibody-drug conjugates (ADC) emerge as promising tools, yet a deeper understanding of antigen dynamics, optimal therapeutic sequencing, and resistance mechanisms is essential. This study investigates the utility of patient-derived xenograft (PDX) models for EC as preclinical platforms, evaluating molecular subtypes and the ADC targets expression of patient and PDX tumors.

**Methods:**

We developed a comprehensive panel of molecularly characterized PDX models from patients with EC representing various histological types. Molecular subtypes and gene alterations were analyzed using sequencing and immunohistochemistry. ADC targets, including human epidermal growth factor receptor 2, trophoblast cell-surface antigen 2, B7-H4, folate receptor alpha, and cadherin-6, were profiled.

**Results:**

Thirty-one EC-PDX models were successfully established, maintaining histological fidelity and 93.1 % molecular subtype consistency with the patient tumors. Notably, 80.6 % of the PDX models exhibited high expression (2+/3+) of at least one ADC target, and 54.8 % displayed high expression of multiple targets. Remarkably, 9.7 % showed high expression of all targets, with gene mutations also characterized. Meanwhile, patient tumors, 78.8 % showed high expression (2+/3+) of at least one ADC target, and 63.6 % showed high expression of multiple targets.

**Conclusion:**

The molecularly classified EC-PDX panel, enriched with detailed antigen profiles and genetic data, provides a robust platform for investigating novel ADC therapies and precision treatment strategies for high-grade EC.

## Highlights

• Established a panel of 31 EC-PDX maintaining histology and achieved 93.1 % molecular subtype consistency with patient tumors.
• Evaluated the expression of five ADC targets-HER2, TROP2, B7-H4, FRα, and CDH6- in both patient tumors and EC-PDX.
• 78.8 % of patient tumors showed high-expression of at least one ADC target, and 63.6 % displayed multiple targets.
• 80.6 % of EC-PDX showed high-expression of at least one ADC target, and 54.8 % displayed multiple targets.
• Comprehensive EC-PDX provides a platform for investigating precision treatment strategies for high-grade EC.

## Background

Endometrial cancer (EC) is the most common gynecological malignancy in developed countries. The incidence rates vary worldwide, with higher rates reported in North America and Europe than in Asia and Africa. In the United States, EC is the most common gynecological cancer, with an estimated 67,880 new cases of EC in 2024 [1]. Approximately 10–15 % of all patients with EC are at an advanced stage, and the 5-year survival rate of patients with distant metastases is reported to be 17 %. The histological type has also been reported as a prognostic factor. Serous, clear cell, and carcinosarcoma subtypes are clinically aggressive and associated with recurrence [2]. Histomorphological evaluation of EC, including high-grade carcinomas, has been reported to be poorly reproducible [2,3].

The Cancer Genome Atlas (TCGA) has been used to comprehensively characterize the genomic alterations in various types of cancer. The TCGA project has provided valuable genomic data on EC, allowing a better understanding of the molecular landscape of the disease. It classified ECs into four molecular subtypes, namely *POLE* ultramutated (POLE, 7 %), microsatellite instability hypermutated (MSI-H, 28 %), copy-number low (CN-L, 39 %), and copy-number high (CN-H, 26 %). All four subtypes are associated with progression-free survival (PFS). Clusters with POLE tumors are the best prognosis and copy CN-H tumors are the worst it [4]. These four subtypes were reflected in the 2020 World Health Organization (WHO) classification are divided with using targeted sequencing of the POLE exonuclease domain, and immunohistochemistry (IHC) which utility for clinical [5]. In the 2020 WHO classification, EC was categorized into *POLE* mutation (POLEmut), mismatch repair-deficient (MMRd), p53 abnormal (p53abn), and no specific molecular profile (NSMP). To know these subtypes of EC, we can reflect to the treatment; POLEmut and MMRd tumors are associated with high numbers of neoantigen loads and tumor-infiltrating lymphocytes (TILs). Immunotherapies, such as programmed cell death protein-1 (PD-1) checkpoint inhibitors, have been suggested for these tumors considering their good response [6]. Although p53abn tumors have the worst prognosis, they currently have no effective treatment.

Antibody-drug conjugates (ADCs) are new complex therapeutics that consist of monoclonal antibodies directed toward tumor-associated antigens, with potent cytotoxic agents such as a payload conjugated with a linker [7]. ADCs form a promising treatment option for patients with gynecological cancers that express many ADC target proteins [8]. In particular, we have shown that a certain percentage of patients with uterine carcinosarcoma and poor prognosis express human epidermal growth factor receptor 2 (HER2), and that trastuzumab deruximab (T-DXd) is effective in both preclinical and clinical settings [9,10]. An ADC against trophoblast cell-surface antigen 2 (TROP2), namely sacituzumab govitecan, showed promising results in a phase II trial for EC [11]. Various ADCs for other targets have been developed for gynecological cancers and are currently in clinical trials.

The patient-derived xenograft (PDX) model is a tumor-bearing mouse model established by directly transplanting patient tumor tissues into immunocompromised mice. The PDX model reflects the microenvironment and heterogeneity of patient tumors and is considered a preclinical model that reflects clinical efficacy more precisely than conventional cell line models. Drug screening for ADCs using the PDX model is considered a highly useful evaluation method owing to the possibility of repeated evaluations, easy availability of tumor tissue after drug administration, and the possibility of contrasting antigen expression in the tumor with drug efficacy [12].

In this study, we successfully constructed 31 EC-PDX models using tumor tissues obtained from 64 consecutive patients with EC. To investigate the utility of these EC-PDX models as preclinical platforms, we evaluated the molecular subtypes of patient and PDX tumors using sequencing and immunohistochemistry (IHC). In addition, genetic mutations, gene amplification, and expression of ADC target proteins were systematically elucidated. The results indicated that our EC-PDX panel is highly useful for drug screening, with confirmed expression of various ADC target proteins across both histological types and molecular subtypes. Drug screening of ADCs using our EC-PDX panel might help develop new therapeutic strategies, elucidate the mechanism of resistance, and deepen our understanding of the molecular biology of EC, which is yet to be clarified.

## Materials and methods

### Patients and ethics statement

This study was performed at the Saitama Medical University International Medical Center (SIMC, Saitama, Japan) and the National Cancer Center Research Institute (NCCRI, Tokyo, Japan) after obtaining institutional review board approval. The subjects were 64 patients with high-grade EC before surgery at the SIMC between June 2016 and June 2019. Of the 64 patients, 63 underwent primary total hysterectomy with or without lymphadenectomy as the primary surgery. One case was a recurrence presenting with ascites. All participants provided written informed consent. All protocols were approved by the institutional review boards (SIMC: 12–096, NCCRI: 2016–121). The study was conducted in alignment with the guidelines of the Declaration of Helsinki, ensuring compliance with all relevant regulations, guidelines, and local policies. All animal experiments adhered to the Institute for Laboratory Animal Research guidelines (NCCRI T19-008).

### Establishment of PDX models

EC-PDX models were established as described previously [10]. Briefly, tumor tissues were immediately collected from patients with EC at the time of surgery. The collected specimens were transplanted subcutaneously into the flanks of 6-week-old female SCID-Beige mice (CB17.Cg-PrkdcscidLystbg-J/CrlCrlj; Japan Charles River Co., Ltd., Kanagawa, Japan). The mice were housed in sterile filter-capped polycarbonate cages, maintained in a barrier facility on a 12-h light/dark cycle, and provided with sterilized food and water. They were monitored weekly for tumor growth. When the tumor grew to approximately 1,000–2,000 mm^3^ (volume calculated using the following formula: tumor volume (mm^3^) = [tumor length × (tumor width)^2^]/2), the tumor was designated as transgeneration 1 (TG1). It was then excised, fragmented into 2-mm cubes, and passaged to mice to create further generations (TG2, TG3, etc.).

### Whole-exome sequencing

Whole-exome sequencing was performed by Almac Diagnostics (Souderton, USA) using FFPE samples from patients and PDX tumors. After hematoxylin and eosin staining, the tumor sites were macro-dissected, and DNA and RNA were extracted using the Qiagen AllPrep DNA/RNA mini kit (Qiagen, Hilden, Germany). DNA processing was performed using the Agilent SureSelect Clinical Research Panel v2 (Agilent, CA, USA), achieving a mean coverage of 100 × . Prior to processing in the main pipeline, raw PDX sequence data in FASTQ format were aligned to a concatenated human-mouse genome. Reads mapped to both human and mouse sequences as well as those mapped exclusively to mouse sequences were discarded. Subsequently, the sequencing reads were aligned to the human reference genome (GRCh37/hg19) using the BWA-MEM algorithm (v0.7.15) and processed using The Genome Analysis Toolkit (GATK, RRID: SCR_001876) Best Practices [13,14]. Duplicate reads were identified using the Picard MarkDuplicates tool (RRID:SCR_006525), followed by base recalibration to correct for patterns of systematic errors in base quality scores [15]. These operations produced clean analysis-ready BAM files for each sample. Somatic variants were identified using Mutect2 (GATK version 4.1.1.0) in the tumor-only mode, following the manufacturer's recommended settings. Almac's proprietary Panel of Normals, comprising of 45 blood samples sequenced using the All Exon V7 Panel, was used in variant calling to capture recurrent technical artifacts. The raw output of Mutect2 was filtered using the GATK4 FilterMutectCalls tool to generate a preliminary set of somatic variants. To produce an accurate set of somatic variants suitable for downstream analysis, variants assigned as “PASS” by FilterMutectCalls were processed further as follows: multi-allelic variants and single-nucleotide variants located less than five bases from the read ends were removed, an optimized germline filtering strategy was applied to remove potential germline variants, and a machine learning-based approach was applied to filter out potential FFPE artifacts. After filtering, call sets were annotated using SnpEff (version 4.3t, RRID:SCR_005191) with genome version GRCh37/hg19 and Ensembl annotation version 75 (RRID:SCR_002344), and supplemented with annotations from COSMIC (release 90, RRID:SCR_002260) and dbSNP (build 151, RRID: SCR_002338).

### Verification of Concordance and Contamination in PDX Model Establishment

To detect sample swaps and cross-individual contamination during the PDX model establishment process, we performed Conpair analysis on paired BAM files (PDX tumor and patient tumor) using a reference genome and a predefined set of genomic markers. This analysis allowed us to verify concordance between the PDX tumor and the patient tumor and to estimate contamination levels [16]. Models with a concordance rate of less than 70 % were considered contaminated or mishandled and were excluded from further analysis.

### Immunohistochemistry (IHC)

Immunostaining was performed using formalin-fixed paraffin-embedded sections from patient and PDX tumors. To evaluate the hematoxylin and eosin (H&E) staining and IHC results, a pathologist examined all the samples. HER2, ER, and p53 staining were performed as described previously [10]. Immunostaining of postmeiotic segregation increased 2 (PMS2) (clone EP51, Dako, Glostrup, Denmark, RRID:AB_3331634), MutS homolog 6 (MSH6) (clone EP49, Dako, RRID:AB_2889975), HER2 (Ventana Pathway HER2 4B5, Ventana/Roche diagnostics, Arizona, USA, RRID: AB_10981779), TROP2 (Clone MAB650, R&D systems, Minneapolis, MN, USA, RRID:AB_2205665), B7-H4 (clone D1M8I, Cell signaling technology, Beverly, MA, USA, RRID: AB_2750878), Folate Receptor alpha (FRα) (Folate Receptor alpha IHC assay Kit 26B3. F2, Biocare Medical, Pacheco, CA, USA), cadherin-6 (CDH6) (HPA007456/rabbit polyclonal, Atlas Antibodies AB, Bromma, Sweden, RRID: AB_1078373), ER (clone SP1; Ventana/Roche Diagnostics, Arizona, USA, RRID: AB_2857956), and p53 (clone DO-7; Dako, Glostrup, Denmark) was conducted. The staining intensity was categorized as follows: "0″ for absent expression, "1+" for weak staining, "2+" for moderate staining, and "3+ for strong staining. In this study, we defined "expression" as IHC score 1+–3+, "low expression" as IHC score 1+, and “high expression" as IHC score above 2+.

Antibodies from Dako/Agilent, including MSH6 (RRID:AB_2889975), PMS2 (RRID:AB_3331634), and p53, were stained using Dako's Automated Staining System (LINK48; Dako, CA, USA), according to the manufacturer's instructions. The ER antibody (Ventana/Roche) was used and stained using Ventana's automated staining system (Benchmark XT). A diffuse pattern of nuclear-positive cells, with at least 80 % of the tumor cells showing p53 positivity, indicated the presence of a *TP53* gene mutation [17]. A mosaic staining pattern with scattered nucleus-positive cells was indicative of the wild type pattern. The diffuse pattern or complete absence of p53 expression (“null pattern”) in EC was considered as the staining pattern suggestive of *TP53* gene mutations [17].

### Molecular subtypes of EC-PDX models

Patient tumors and EC-PDX models are categorized into four non-overlapping molecular subtypes, namely (i) those harboring mutations in the gene encoding the exonuclease domain of DNA polymerase epsilon (POLEmut), (ii) those exhibiting loss of staining for either PMS2 or MSH6 (MMRd), (iii) those with a diffuse or null staining pattern in p53 immunostaining (p53abn), and (iv) those lacking any of these three molecular abnormalities, a diagnosis of exclusion known as NSMP. This study classified double-/dual-classifier EC into upstream branching groups as reported previously [18]**.**

### Statistical analysis

Differences among the groups of patients were assessed via one-way analysis of variance, Student's t-test, or chi-square test. Significant differences between groups were identified using the log-rank test. Survival analyses employed the Kaplan–Meier estimator method. Statistical analyses were conducted using GraphPad Prism 10 software (GraphPad Software, San Diego, CA, USA, RRID:SCR_002798). All p-values reported are two-sided, with *p* < 0.05 considered statistically significant.

### Data availability

The datasets utilized and/or analyzed in this study are accessible from the corresponding author upon reasonable request.

## Results

### Establishment of EC-PDX models

We successfully established 31 EC-PDX models (47.7 %) using 65 samples collected from 64 patients. Although 34 EC-PDX models were initially established, three PDX models (derived from EC-Pt 11, 20, and 25) were excluded due to potential contamination and were not included in the PDX analysis. The characteristics of the patients from whom EC-PDX models were established are summarized in Table 1. In one case (EC-Pt 15), a PDX model was established from the surgical tissue of the patient's primary tumor and from ascites cells after recurrence. Supplementary Table 1 shows the differences in clinical backgrounds across the patients from whom a EC-PDX model could be established and those from whom it could not. Although there was no clear difference in age or stage between the two groups, a higher PDX establishment rate was observed in patients with positive lymphovascular space invasion (LVSI) at the time of surgery. Additionally, the success rate of PDX establishment was higher in carcinosarcoma (10 of 15 samples, 66.7 %). In terms of the association with patient survival, patients from whom PDX could be established tended to have shorter progression-free survival (PFS) and overall survival (OS) than those from whom PDX could not be established (Supplementary Figure 1).

**Table 1:** The characteristics of the patients from whom EC-PDX models were established initially.

EC-Pt No.	EC-PDX No.	Age	FIGO stage	Histology	Sampling site	Sampling point
1	1	59	II	Endometrioid G2	Endometrial tumor	Primary surgery
2	2	69	IIIC2	Adenocarcinoma unclassified ^a)^	Endometrial tumor	Primary surgery
3	3	62	IIIC1	Endometrioid G3	Endometrial tumor	Primary surgery
4	4	55	IIIC2	Carcinosarcoma	Endometrial tumor	Primary surgery
5	5	58	IIIB	Endometrioid G3 ^b)^	Endometrial tumor	Primary surgery
6	6	78	IVB	Carcinosarcoma	Endometrial tumor	Primary surgery
7	7	49	IIIC1	Mixed ^c)^	Endometrial tumor	Primary surgery
8	8	75	IA	Serous	Endometrial tumor	Primary surgery
9	9	63	IA	Mixed ^d)^	Endometrial tumor	Primary surgery
10	10	52	IIIA	Endometrioid G3	Endometrial tumor	Primary surgery
11	11	74	IB	Endometrioid G1	Endometrial tumor	Primary surgery
12	12	62	IA	Serous	Endometrial tumor	Primary surgery
13	13	65	IIIC2	NEC	Endometrial tumor	Primary surgery
14	14	58	II	Undifferentiated	Endometrial tumor	Primary surgery
15	15	58	IVB	Carcinosarcoma	Endometrial tumor	Primary surgery
	15-2	58	IVB	Carcinosarcoma	Ascites cells	Recurrence
16	16	57	IIIB	Endometrioid G3	Endometrial tumor	Primary surgery
17	17	68	IB	Carcinosarcoma	Endometrial tumor	Primary surgery
18	18	76	IIIA	Serous	Endometrial tumor	Primary surgery
19	19	67	IIIC2	Serous	Endometrial tumor	Primary surgery
20	20	74	II	Carcinosarcoma	Endometrial tumor	Primary surgery
21	21	68	IVB	Carcinosarcoma	Endometrial tumor	Primary surgery
22	22	74	IB	Serous	Endometrial tumor	Primary surgery
23	23	62	IB	Carcinosarcoma	Endometrial tumor	Primary surgery
24	24	65	IA	Clear cell	Endometrial tumor	Primary surgery
25	25	65	IB	Endometrioid G1	Endometrial tumor	Primary surgery
26	26	56	IB	Carcinosarcoma	Endometrial tumor	Primary surgery
27	27	57	IIIB	Endometrioid G3	Endometrial tumor	Primary surgery
28	28	55	IIIB	Endometrioid G3	Endometrial tumor	Primary surgery
29	29	73	IB	Carcinosarcoma	Endometrial tumor	Primary surgery
30	30	55	IA	Serous	Endometrial tumor	Primary surgery
31	31	49	II	Endometrioid G2	Endometrial tumor	Primary surgery
32	32	73	IB	Endometrioid G3	Endometrial tumor	Primary surgery
33	33	68	IVB	Carcinosarcoma	Ascites cells	Recurrence

Abbreviation: NEC, neuroendocrine carcinoma.

a) Most likely Endometrioid carcinoma G3, b) with squamous differentiation c) Serous and clear cell carcinoma, d) Endometrioid G3 and clear cell carcinoma.

### EC-PDX models maintained the original histology and molecular classification

**Fig. 1:**
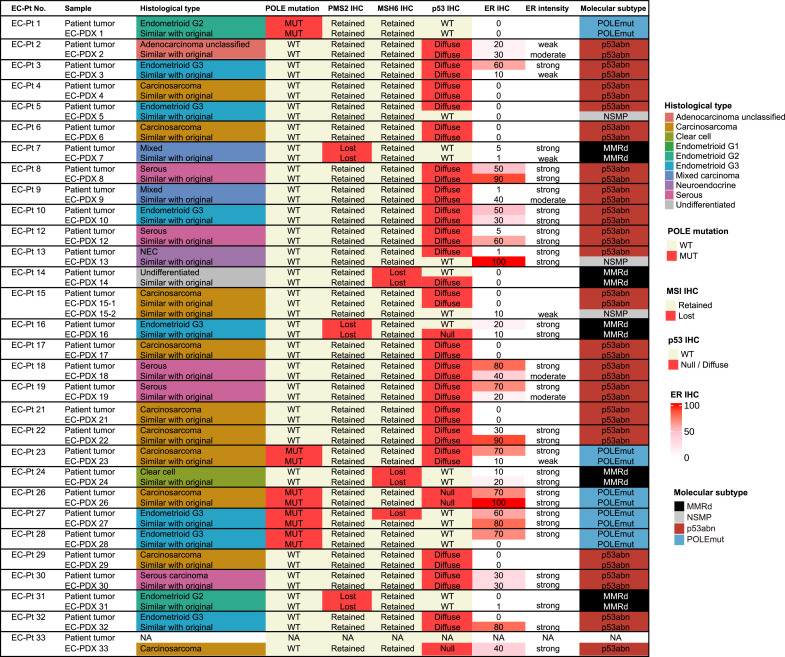
**Comparison of the histomolecular classification between patient tumors and PDX models.** NEC, neuroendocrine carcinoma; MUT, mutation-positive; WT, wild type; POLEmut, POLE mutation; MMRd, mismatch repair deficiency; p53abn, p53 abnormal; NSMP, non-specific molecular profile. a) Most likely endometrioid carcinoma G3, b) with squamous differentiation, and c) serous and clear-cell carcinoma, d) endometrioid G3 and clear cell carcinoma.

**Fig. 2:**
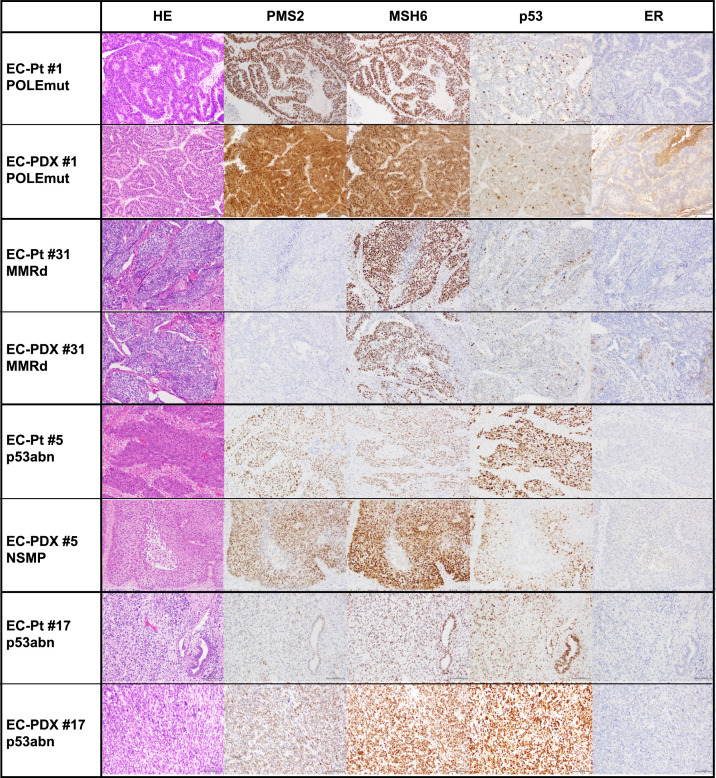
**Representative images of the cases in each molecular subtype.** Histological and molecular subtype findings of the original and patient-derived xenograft (PDX) tumors. POLEmut, *POLE*mutation; MMRd, mismatch repair deficiency; p53abn, p53 abnormal; NSMP, no specific molecular profile.

As shown in Fig. 1, among the 31 EC-PDX models derived from 30 patients, 9 (29.0 %) were endometrioid, six (19.4 %) were serous, one (3.2 %) was clear cell, 10 (32.3 %) were carcinosarcoma, and five (16.1 %) were other carcinomas. The histological types of all PDX tumors were confirmed to be similar to those of the patient tumors (Fig. 2). For molecular subtypes, the EC-PDX models comprised of five *POLE*mut, five MMR-d, 18 p53abn, and three NSMP tumors. In 29 primary patient tumor-PDX pairs, 93.1 % (27 out of 29 pairs) of PDX tumors maintained the molecular subtype of the patient tumor while two models (EC-PDX 5 and 13) showed changed molecular classification, p53abn to NSMP.

### Evolution of ADC target antigens: Patient tumors vs. PDX models

We evaluated the expression of antigens, recognized as potential therapeutic targets of ADCs, which were expected to be effective in EC treatment, including HER2, TROP2, B7-H4, FRα, and CDH6, in both PDX models and the patient tumors. Supplementary Figure 2 shows the representative IHC images of ADC targets across different molecular subtypes. As shown in Supplementary Figure 3, the concordance of ADC targets between the patient tumors and PDX models (IHC score of 0 vs.1–3+) was 70.0 %, 73.3 %, 60.0 %, 60.0 %, and 90.0 % for HER2, TROP2, B7-H4, FRα, and CDH6, respectively. CDH6 showed consistent expression while B7-H4 and FRα displayed more variability. The rate of no-expression of HER2, B7-H4, and FRα was increased in PDX tumors than in primary tumors.

### Intra-tumor interplay and cross-subtype distribution of multiple ADC targets

**Fig. 3:**
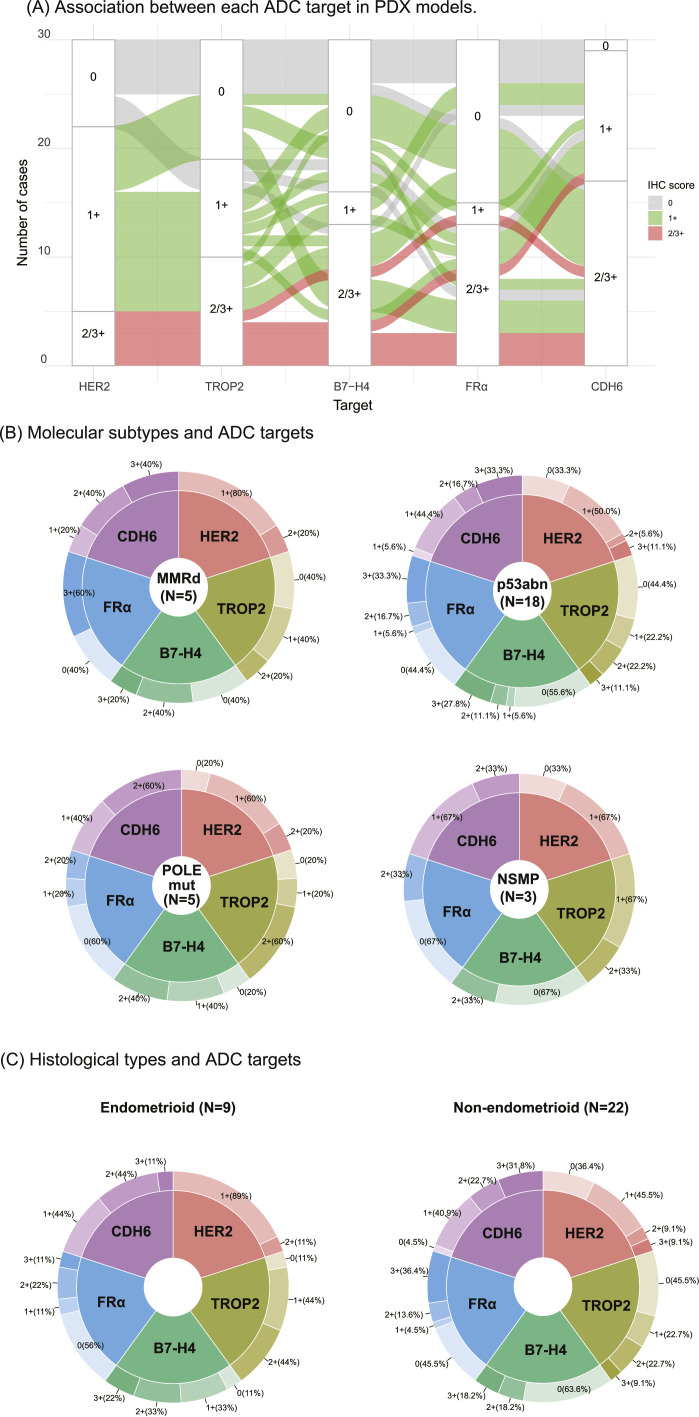
**Demographics of ADC targets in EC-PDX models.** IHC, Immunohistochemistry; POLEmut, *POLE*mutation; MMRd, mismatch repair deficiency; p53abn, p53 abnormal; NSMP, no specific molecular profile. (A) Association between each ADC target in EC-PDX model. (B) Molecular subtypes and ADC targets. (C) Histological types and ADC targets.

To assess the intra-tumor interactions of multiple ADC target proteins and their distribution across molecular subtypes, the results for PDX models and patient tumors are presented in Fig. 3 and Supplementary Figure 4, respectively. Table 2 and Fig. 3A summarize the expression of ADC targets and their overlaps with the PDX models. Among the 31 PDX models, 25 (80.6 %) showed high expression (2+/3+) of at least one target and 17 (54.8 %) demonstrated high expression of multiple targets. High expression of all five targets was observed in three PDX models (9.7 %), and only one model lacked the expression of all five targets. B7-H4 and FRα were found to not be expressed frequently than the other targets in PDX models, whereas CDH6 was more commonly expressed in PDX models. HER2 expression was often lower than that of the other targets; however, 88.9 % (16 out of 18) of the HER2 1+-expressing PDX models displayed high expression of the other ADC targets.

**Table 2:** Expression of therapeutic targets in EC-PDX models.

EC-PDX No.	Histological type	Molecular Subtype	HER2	TROP2	B7-H4	FRα	CDH6
**1**	Endometrioid G2	*POLE*mut	1+	2+	1+	2+	1+
**2**	Adenocarcinoma unclassified ^a)^	p53abn	1+	2+	0	0	3+
**3**	Endometrioid G3	p53abn	1+	2+	1+	0	1+
**4**	Carcinosarcoma	p53abn	0	0	0	0	1+
**5**	Endometrioid G3 ^b)^	NSMP	1+	1+	0	0	2+
**6**	Carcinosarcoma	p53abn	0	0	0	0	1+
**7**	Mixed ^c)^	MMRd	1+	1+	0	3+	1+
**8**	Serous	p53abn	0	1+	0	3+	2+
**9**	Mixed ^d)^	p53abn	3+	2+	3+	3+	3+
**10**	Endometrioid G3	p53abn	1+	2+	3+	0	2+
**12**	Serous	p53abn	1+	0	2+	3+	3+
**13**	NEC	NSMP	0	1+	2+	0	1+
**14**	Undifferentiated	MMRd	1+	0	0	0	3+
**15-1**	Carcinosarcoma	p53abn	0	1+	0	2+	1+
**15-2**	Carcinosarcoma	NSMP	1+	2+	0	2+	1+
**16**	Endometrioid G3	MMRd	1+	0	2+	3+	3+
**17**	Carcinosarcoma	p53abn	1+	0	0	1+	1+
**18**	Serous	p53abn	3+	3+	2+	3+	1+
**19**	Serous	p53abn	1+	1+	0	0	3+
**21**	Carcinosarcoma	p53abn	0	0	0	0	1+
**22**	Serous	p53abn	2+	3+	3+	3+	3+
**23**	Carcinosarcoma	*POLE*mut	0	0	0	0	2+
**24**	Clear cell	MMRd	2+	2+	3+	3+	2+
**26**	Carcinosarcoma	*POLE*mut	1+	2+	2+	0	2+
**27**	Endometrioid G3	*POLE*mut	2+	2+	2+	1+	2+
**28**	Endometrioid G3	*POLE*mut	1+	1+	1+	0	1+
**29**	Carcinosarcoma	p53abn	0	0	0	0	0
**30**	Serous	p53abn	1+	0	3+	2+	2+
**31**	Endometrioid G2	MMRd	1+	1+	2+	0	2+
**32**	Endometrioid G3	p53abn	1+	1+	3+	2+	1+
**33**	Carcinosarcoma	p53abn	1+	0	0	3+	3+

Abbreviation: NEC, neuroendocrine carcinoma; MUT, mutation positive; WT, wild-type; *POLE*mut, *POLE* mutation; MMRd, mismatch repair deficiency; p53abn, p53 abnormal; NSMP, no specific molecular profile.

a) Most likely Endometrioid carcinoma G3, b) with squamous differentiation c) Serous and clear cell carcinoma, d) Endometrioid G3 and clear cell carcinoma.

To further evaluate the expression status of ADC targets across the molecular subtypes and histologies, we generated models, as shown in Fig. 3B and 3C. We found that the expression patterns were largely similar across the four molecular subtypes. Notably, in p53abn tumors, which are in need of further therapeutic development due to their poor prognosis, the expression rates of therapeutic targets, excluding B7-H4, are above 50 %. Additionally, the frequencies of high expression of TROP2, FRα, and CDH6 exceeded 30 %. In contrast, NSMP tumors exhibited lower frequencies of high expression across all therapeutic targets than other molecular subtypes. Furthermore, 3+ expression was more frequently observed in non-endometrioid tumors than in endometrioid tumors.

For patient tumors, we summarized the overlap and characteristics of antigen expression in Supplementary Figure 4A and Supplementary Table 2. Of the 33 cases, 26 (78.8 %) had at least one antigen with high expression and 21 (63.6 %) exhibited high expression of more than two antigens. High expression across all five targets was observed in three cases (9.1 %), whereas only one case (3.0 %) showed no expression of any of the five targets. These findings suggested the potential for complementary antigen expression in individual cases. As shown in Supplementary Figures 4B and 4C, the antigen expression patterns were largely similar across the *POLE*mut, MMR-d, and p53abn subtypes, although there was only one case of the NSMP subtype. Additionally, we found that 3+ expression of HER2, FRα, and CDH6 was more frequently observed in non-endometrioid tumors than in endometrioid tumors, consistent with the findings from PDX models.

### Genetic alterations in EC-PDX models

**Fig. 4:**
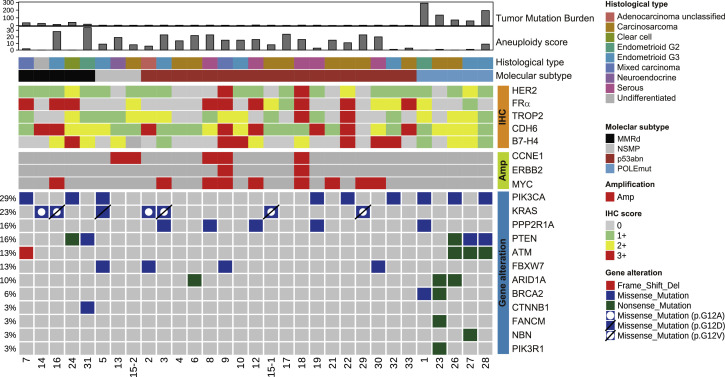
**Genomic characteristics and the expression status of therapeutic targets of all EC-PDX models that are grouped by the four molecular subtypes.** POLEmut, *POLE*mutation; MMRd, mismatch repair deficiency; p53abn, p53 abnormal; NSMP, no specific molecular profile.

Somatic mutations and gene amplification status in the EC-PDX models are shown in Fig. 4. As expected, *POLE*mut tumors exhibited a higher tumor mutation burden (TMB) than the other subtypes, with notable mutations in *PIK3CA* (60.0 %) and *PTEN* (60.0 %). MMRd tumors showed a high frequency of *KRAS* mutations (40.0 %) than the other subtypes. In p53abn tumors, mutations in *PPP2R1A* (22.2 %) and *FBXW7* (16.7 %) were observed, although the mutations were not found in other molecular classifications. Additionally, p53abn tumors tended to exhibit amplification of *CCNE1* (16.7 %), *ERBB2* (11.1 %), and *MYC* (44.4 %) genes.

## Discussion

In this study, 65 tumor samples from 64 patients were used to generate 31 EC-PDX models. Each model reflected the histological and molecular characteristics of the corresponding patient tumor, and the EC-PDX panel encompassed all four molecular subtypes essential for therapeutic strategies in EC. Through this analysis, we characterized the expression of ADC target proteins in patient tumors and their corresponding PDX models and examined the co-expression of multiple ADC target proteins within each tumor. The findings provided valuable foundational insights for selecting ADCs for EC treatment, thereby shaping future therapeutic strategies.

In total, 31 PDX models were generated from 65 tumor tissues in this study, with a success rate of 47.7 %. In our previous experience with PDX library construction, the average success rate was approximately 30 % across all cancer types [19]. Previous reports on EC-PDX establishment success rates ranged from 18 to 100 %, suggesting that relatively high success rates may be achieved with high-grade EC [20]. Successful establishment of PDX tends to be associated with worse prognosis and a higher tumor grade than unsuccessful establishment, suggesting that more rapidly growing tumors may be successfully established in PDX [21]. Globally, EC-PDX models are limited in number than that of other cancer types, with the 24 models reported by Depreeuw et al. being the most common [22]. CancerModels.org is a global open catalog of harmonized patient-derived cancer models; however, it has only 31 EC-PDX models out of a total of 5,266 models, making our EC-PDX panel a very valuable non-clinical model platform [23].

The 31 EC-PDX models established in this study faithfully recapitulated the histological and molecular subtypes of the tumors. In particular, the molecular subtypes were consistent in >90 % of the models; hence, this is the first report to compare the molecular subtypes of patient tumors and PDX models. Molecular subtypes of EC have recently been included in the WHO tumor classification [5] and the new International Federation of Gynecology and Obstetrics (FIGO) staging system [24]. Currently, to provide more effective and less toxic adjuvant treatment strategies based on molecular subtypes, the RAINBOW program is conducting international clinical trials and comprehensive research on each molecular subtype [25]. Among these, p53abn has the poorest prognosis and requires aggressive therapeutic development. Our EC-PDX panel included 18 models (58.1 %) of p53abn, and we believe that we have established an important preclinical platform for p53abn EC. Research on acceleration of drug discovery and development using our EC-PDX panel is warranted.

Drug discovery and development of ADCs have increased at an accelerated rate in recent years, with >100 drugs currently being used in clinical trials [26]. Although comprehensive genetic analyses of EC have been conducted, driver gene mutations, such as EGFR mutations in non-small cell lung cancer, are yet to be identified, and cytotoxic anticancer drugs remain the key drugs. However, the fact that many ADC target proteins are expressed in EC, perhaps not as drivers but as passengers, is now clear, and the development of many ADCs is progressing at an accelerated speed. To this end, we performed a comprehensive assessment of ADC target expression using the EC-PDX panel. We found that >90 % of the samples showed at least one protein expression from among HER2, TROP2, B7-H4, FRα, and CDH6 in both patient tumors and PDX. Since T-DXd has recently shown efficacy even in HER2-low uterine carcinosarcoma, we would expect efficacy via the bystander effect, even if the ADC target expression is negligible [9]. Thus, our data suggested that ECs are promising targets for ADCs.

Drug development for the ADC targets in EC has been progressing steadily. In particular, the development of T-DXd for HER2 is leading the way, and we recently demonstrated its efficacy in HER2-expressing uterine carcinosarcoma in both an investigator-initiated phase II clinical trial and a co-clinical study using a PDX model [10]. DESTINY-PanTumor02 has also demonstrated tumor-agnostic efficacy in HER2-expressing solid tumors, including EC [27], and is expected to be approved for EC in the future. TROP2, B7-H4, and FRα have also been reported to be highly expressed in 65–95 % [28,29], 30 % [30], and 50 % or more [31] of EC, respectively. Sacituzumab govitecan-hziy [11] for TROP2, AZD8205 [32], SGN-B7H4 V [33], and XMT-1660 [34] for B7-H4, and falretuzumab ectoeribulin [35], mirvetuximab soravatansine [36] for FRα, and the results are awaited. High CDH6 expression has been reported in ovarian cancer [37]. However, data regarding its prevalence in EC is scarce. Our data showed that CDH6 is expressed in >80 % of both patient tumors and PDX, and there were many cases in which CDH6 was expressed even if other ADC targets were negative. With the development of ADCs further, multiple ADCs for the same disease and multiple ADCs for the same target might be expected to be available. In this context, the overlap in ADC target expression, identified in this study, would be important for drug development and construction of therapeutic strategies. In addition, when multiple ADC targets are expressed, it is important to determine the treatment sequence and how to select the next ADC in case of resistance to any particular. Our EC-PDX panel enabled us to evaluate these clinically important issues in a non-clinical setting and can possibly be utilized in the development of ADCs.

In this study, we evaluated the EC-PDX panel for genetic abnormalities and found that the four molecular subtypes maintained the same genetic characteristics as reported previously [4]. Notably, we found amplification of *CCNE1, ERBB2,* and *MYC* genes in p53abn, a subtype with a particularly poor prognosis. As for ADC targets, all p53abn models showed expression of any of the five targets evaluated in this study, and 10/18 models (55.6 %) showed strong expression of two or more targets, suggesting that the biomarker data may contribute to the improvement of p53abn prognosis. Recently, Jamieson et al. reported the results of shallow whole-genome sequencing of 187 p53abn ECs and concluded that 75 % of patients had some kind of targetable gene alteration, indicating the need for patient stratification and the development of therapeutic strategies [38]. Our EC-PDX panel supported their findings, and the expression status of ADC targets could be valuable in the development of therapeutic strategies for p53abn. Further studies using our EC-PDX panel would be required to improve the prognosis of EC, especially p53abn.

This study has several limitations. In some cases, subtypes were altered in the four PDX models than in the patient tumors. This could partly be because EC tumors are composed of multiple complex subclonal cell populations, leading to heterogeneity within the tumor. Secondly, our EC-PDX panel tended to contain more p53abn and less NSMP. This could partly be because p53abn tumors are more aggressive and have a higher success rate of establishment. Overall, we succeeded in generating 31 EC-PDX models and believe that we have established an important platform for preclinical research on EC worldwide. Finally, most studies used primary surgical tissue while only two models used PDX at recurrence. For drug development for advanced recurrent EC, further enhancement of the PDX models at recurrence would be required.

In conclusion, our comprehensive EC-PDX panel faithfully recapitulated not only the molecular subtypes but also the expression of target proteins of ADC and gene alterations. Our findings and other preclinical studies using the models might contribute to future clinical development of each molecular subtype of EC, especially p53abn EC with the poorest prognosis.

## Ethics approval and consent to participate

This study was performed in accordance with the Declaration of Helsinki and was reviewed and approved by the institutional review boards of SIMC and NCCRI (SIMC: 12–096, NCCRI: 2016–121). Written informed consent for participation in the study and publication of the report was obtained from all the participants.

## Funding information

This study was partly supported by Eisai Co. Ltd. and the National Cancer Center Research and Development Fund [grant numbers 2022-A-03, 2023-J-02], Japan.

## CRediT authorship contribution statement

**Sho Sato:** Data curation, Formal analysis, Investigation, Resources, Validation, Writing – original draft, Writing – review & editing. **Shigehiro Yagishita:** Data curation, Formal analysis, Investigation, Resources, Software, Validation, Visualization, Writing – original draft, Writing – review & editing. **Hiroshi Yoshida:** Data curation, Formal analysis, Resources, Supervision, Visualization, Writing – original draft. **Daisuke Shintani:** Investigation, Resources. **Aiko Ogasawara:** Investigation, Resources. **Tadaaki Nishikawa:** Investigation, Resources. **Masanori Yasuda:** Resources, Visualization. **Keiji Furuuchi:** Conceptualization, Supervision. **Toshimitsu Uenaka:** Conceptualization, Supervision. **Akinobu Hamada:** Conceptualization, Funding acquisition, Project administration, Resources, Writing – original draft, Writing – review & editing. **Kosei Hasegawa:** Conceptualization, Project administration, Resources, Supervision, Writing – original draft, Writing – review & editing.

## Declaration of competing interest

The authors declare the following financial interests/personal relationships which may be considered as potential competing interests:

Shigehiro Yagishita declares consulting fee (advisory board) from MSD. Tadaaki Nishikawa declares receipt of an institutional grant from AstraZeneca, honoraria from AstraZeneca and Takeda, speaker's bureau from AstraZeneca, Chugai, Eisai, Genmab, MSD, Roche Diagnostics, Sanofi, Takeda, and Tsumura. Keiji Furuuchi and Toshimitsu Uenaka are employees of Eisai Inc. Akinobu Hamada declare receipt of an institutional grant from Eisai Inc. for this work, institutional grants from AstraZeneca, Eisai, CIMIC, Tosoh, Chordia Therapeutics, Mediford, Eli Lilly, Chugai, Sysmex, Healios, Konica Minolta, and Boehringer Ingelheim outside of this work. Kosei Hasegawa declares institutional contracted research from Daiichi Sankyo, Eisai, MSD, and Takeda outside of this work, consulting fees (advisory board) from Chugai, Eisai, Takeda, MSD, Roche, Genmab, Sanofi, GSK, and Zymeworks outside of this work, honoraria from, Daiichi Sankyo, AstraZeneca, Chugai, Eisai, Genmab, MSD, Takeda, Sanofi, Kyowa Kirin, Kaken, and GSK, support for travel expenses from Regeneron and Seagen.

## Appendix Supplementary materials

Image, application 1

Image, application 2

Image, application 3

Image, application 4

Image, application 5

Image, application 6
