# Supplementary figures and images for: Establishing a comprehensive panel of patient-derived xenograft models for high-grade endometrial carcinoma: molecular subtypes, genetic alterations, and therapeutic target profiling
Source: Neoplasia. 2025 Apr 7;64:101158. doi: 10.1016/j.neo.2025.101158 (PMC12004378; doi:10.1016/j.neo.2025.101158)

(A) Progression-free survival

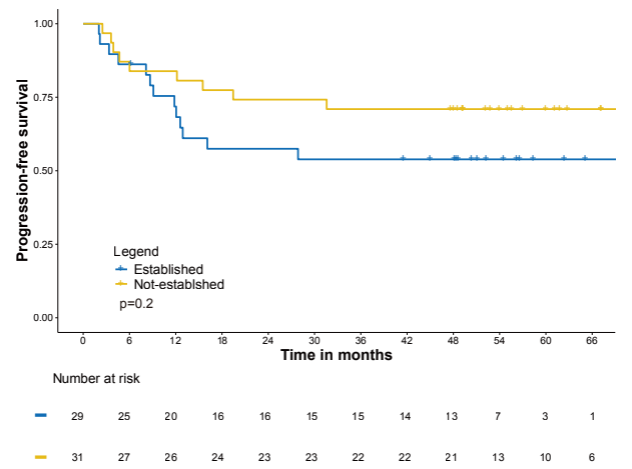

(B) Overall survival

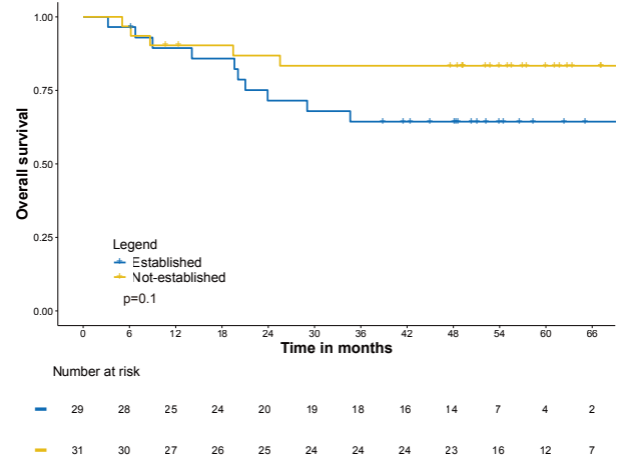

Supplement: Supplementary file 1 [file mmc1.pdf]

Supplementary Figure 3. Association of target expression between patient tumor and PDX tumor.

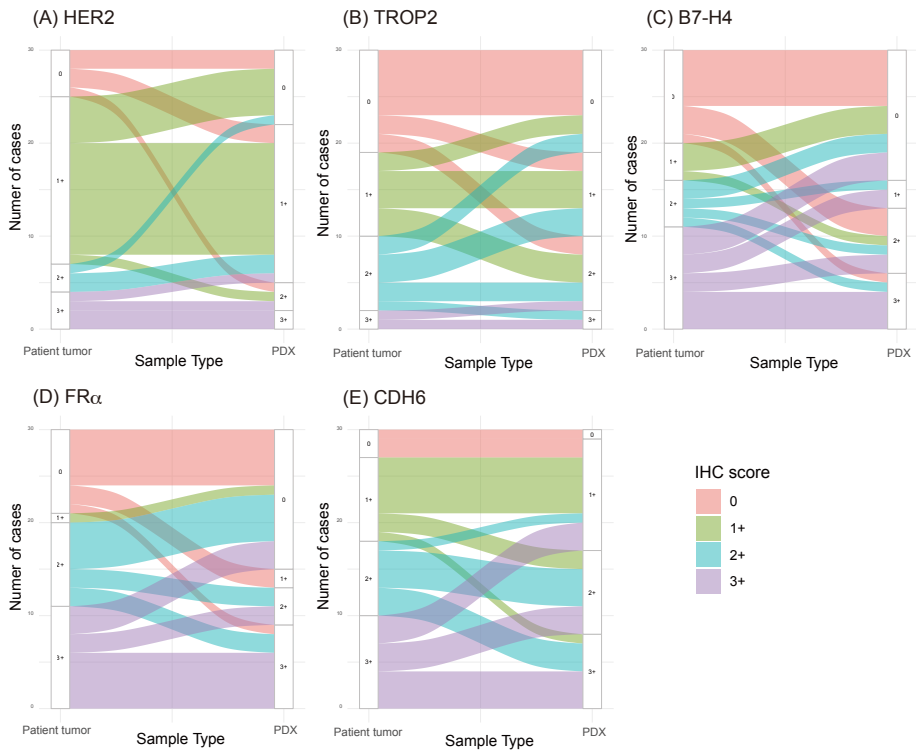

Supplement: Supplementary file 3 [file mmc3.pdf]
