# Supplementary material for: Establishing a comprehensive panel of patient-derived xenograft models for high-grade endometrial carcinoma: molecular subtypes, genetic alterations, and therapeutic target profiling
Source: Neoplasia. 2025 Apr 7;64:101158. doi: 10.1016/j.neo.2025.101158 (PMC12004378; doi:10.1016/j.neo.2025.101158)
Supplement: Supplementary file 2 [file mmc2.pdf]

Supplementary Figure 2.

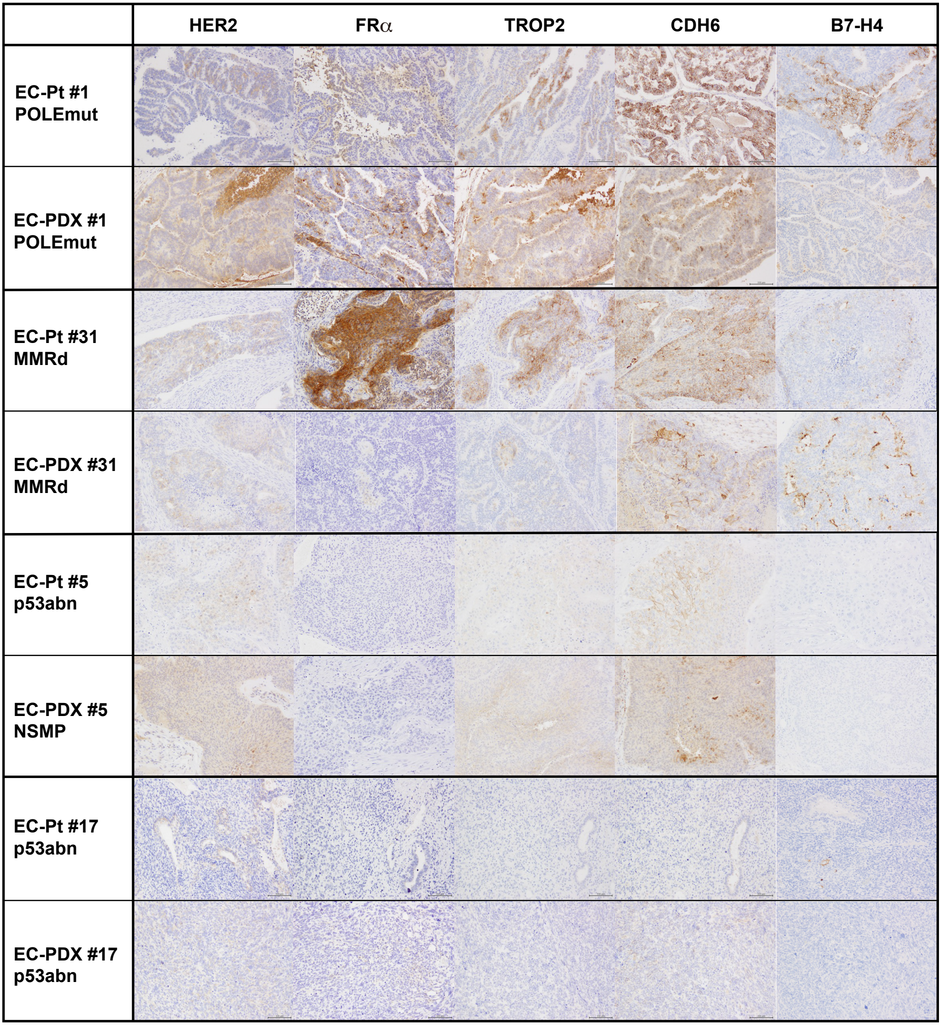

Legend: Therapeutic targets findings of patient and PDX tumors.

Abbreviation: POLEmut, POLEmutation; MMRd, mismatch repair deficiency; p53abn, p53 abnormal; NSMP, no specific molecular profile.
