# Supplementary material for: Establishing a comprehensive panel of patient-derived xenograft models for high-grade endometrial carcinoma: molecular subtypes, genetic alterations, and therapeutic target profiling
Source: Neoplasia. 2025 Apr 7;64:101158. doi: 10.1016/j.neo.2025.101158 (PMC12004378; doi:10.1016/j.neo.2025.101158)

Supplementary Figure 4. Demographic of ADC targets in patient tumor.

(A) Association between each ADC target in patient tumor.

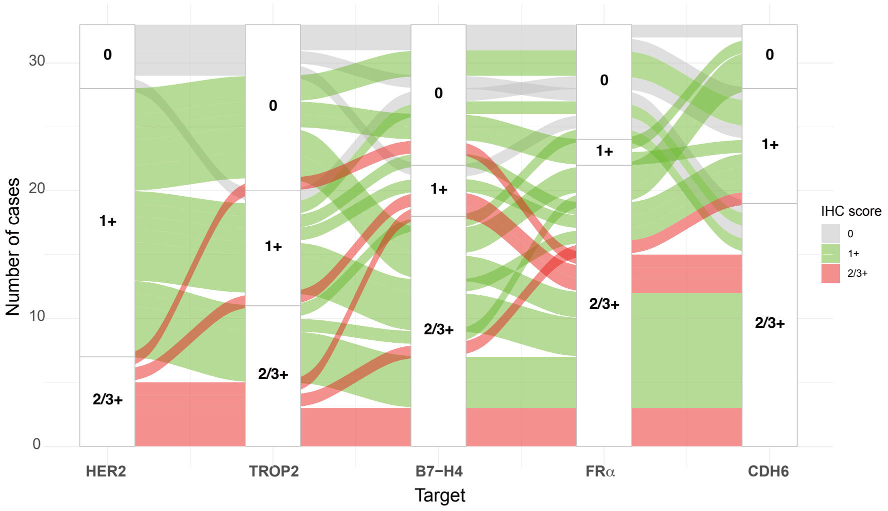

(B) Molecular subtypes and ADC targets

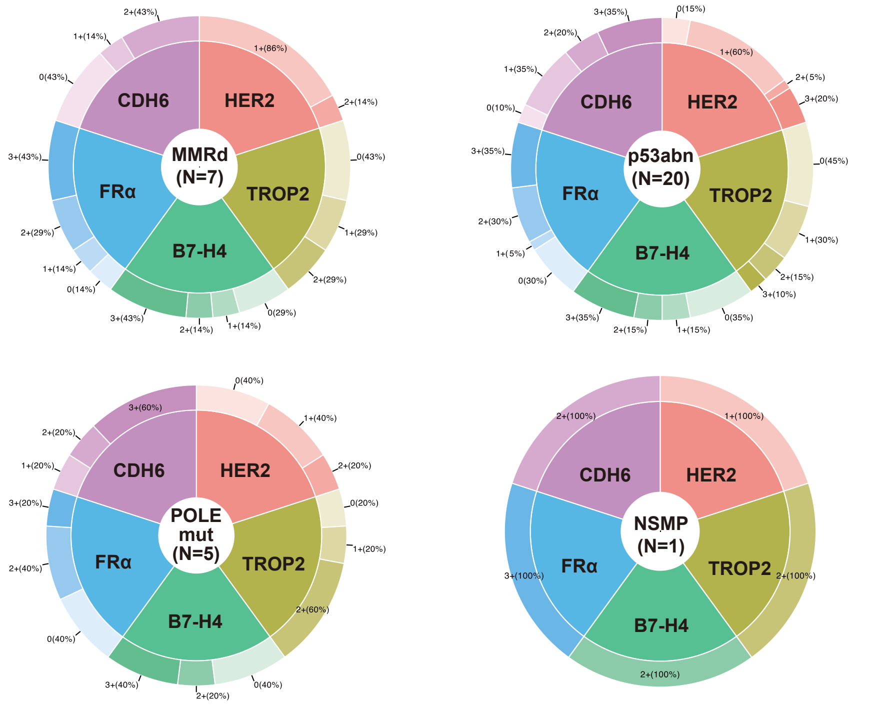

(C) Histological types and ADC targets

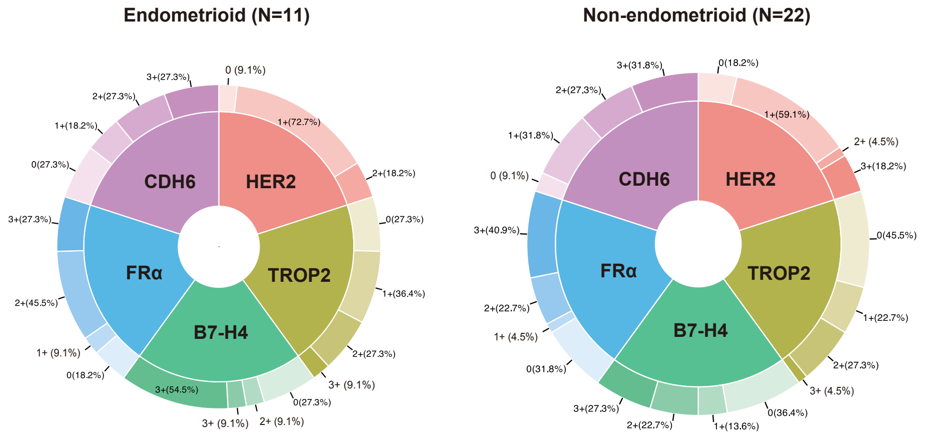

Supplement: Supplementary file 4 [file mmc4.pdf]
