# Supplementary material for: Establishing a comprehensive panel of patient-derived xenograft models for high-grade endometrial carcinoma: molecular subtypes, genetic alterations, and therapeutic target profiling
Source: Neoplasia. 2025 Apr 7;64:101158. doi: 10.1016/j.neo.2025.101158 (PMC12004378; doi:10.1016/j.neo.2025.101158)
Supplement: Supplementary file 5 [file mmc5.pdf]

Supplementary Table 1. The difference of clinicopathological features in between patients with established EC PDX model or patients with non-established.

**Characteristic**	**Established***, N = 31[1]	**Not-establshed**, N = 31[1]	**p-value**[2]
Age	63 (57, 68)	68 (57, 71)	0.9
Histology			0.2
EndometrioidG1/G2	2 (6.5%)	9 (29.0%)	
EndometrioidG3	7 (22.5%)	8 (25.8%)	
Serous	6 (19.4%)	4 (12.9%)	
Clear cell	1 (3.2%)	1 (3.2%)	
Carcinosarcoma	10 (32.3%)	4 (12.9%)	
Others	5 (16.1%)	5 (16.1%)	
Stage			0.4
1	11 (35.4%)	16 (51.6%)	
2	3 (9.7%)	1 (3.2%)	
3	12 (38.7%)	12 (38.7%)	
4	3 (9.7%)	2 (6.5%)	
Recurrence	2 (6.5%)	0 (0%)	
LVSI**			0.05
Negative	6 (19.4%)	14 (45.2%)	
Positive	23 (74.1%)	17 (55.8%)	
Unknown;Recurrence ascites	2 (6.5%)	0	
Adjuvant chemotherapy**			0.05
Received	24 (77.4%)	18 (58.1%)	
Not-received	5 (16.1%)	13 (41.9%)	
Unknown;Recurrence ascites	2 (6.5%)	0	
Samping point			0.5
Primary surgery	29 (93.5%)	31 (100%)	
Recurrence	2 (6.5%)	0 (0%)	

*The three EC-PDXs with possible contamination were not included in the Established group.

** Recurrence ascites cases were not included analysis

[1]Median (IQR); n (%)

[2]Wilcoxon rank sum test; Fisher's exact test; Pearson's Chi-squared test
